# Cold Atmospheric Plasma Inhibits HIV-1 Replication in Macrophages by Targeting Both the Virus and the Cells

**DOI:** 10.1371/journal.pone.0165322

**Published:** 2016-10-26

**Authors:** Olga Volotskova, Larisa Dubrovsky, Michael Keidar, Michael Bukrinsky

**Affiliations:** 1 Department of Mechanical and Aerospace Engineering, The George Washington University, SEAS, Washington, DC, United States of America; 2 Department of Microbiology, Immunology & Tropical Medicine, The George Washington University, SMHS, Washington, DC, United States of America; QIMR Berghofer Medical Research Institute, AUSTRALIA

## Abstract

Cold atmospheric plasma (CAP) is a specific type of partially ionized gas that is less than 104°F at the point of application. It was recently shown that CAP can be used for decontamination and sterilization, as well as anti-cancer treatment. Here, we investigated the effects of CAP on HIV-1 replication in monocyte-derived macrophages (MDM). We demonstrate that pre-treatment of MDM with CAP reduced levels of CD4 and CCR5, inhibiting virus-cell fusion, viral reverse transcription and integration. In addition, CAP pre-treatment affected cellular factors required for post-entry events, as replication of VSV-G-pseudotyped HIV-1, which by-passes HIV receptor-mediated fusion at the plasma membrane during entry, was also inhibited. Interestingly, virus particles produced by CAP-treated cells had reduced infectivity, suggesting that the inhibitory effect of CAP extended to the second cycle of infection. These results demonstrate that anti-HIV activity of CAP involves the effects on target cells and the virus, and suggest that CAP may be considered for potential application as an anti-HIV treatment.

## Introduction

Cold atmospheric plasma (CAP) is a specific form of ionized gas defined as non-thermal non-equilibrium plasma, i.e. a quazi-neutral gas with fast electrons and relatively slow ions. CAP has a complex chemical composition and some of the charged particles detected in the CAP, such as NO^-^, NO_2_^-^, OH^-^, O^-^, as well as reactive oxygen species (O_3,_ O), have important biological and biomedical activities. The ongoing research suggests that CAP can be used for decontamination and sterilization, and appears to be quite effective in virus inactivation [1–4]. The mechanisms behind the anti-viral activity of CAP are poorly understood, but may include both a direct effect on viral particles via bioactive ions and indirect suppression via modification of cell surface receptors important for viral replication [5].

In this study, we investigated the CAP effects on replication of HIV-1. Natural targets of HIV-1 are myeloid cells and CD4-positive T lymphocytes. Since we used plasma jet to produce CAP, we performed this study on adherent monolayers of monocyte-derived macrophages to eliminate variability associated with treatment of suspension cultures of CD4+ T cells. Indeed, careful quantification of the CAP effects in suspension cultures would be difficult due to a multilayer composition of such cultures, which shields lower level cells from irradiation. Here, we report for the first time that CAP treatment induces a suppression of the HIV-1 replication in macrophages by reducing virus-cell fusion and infectivity of the virus produced by treated cells. These data can be further exploited to develop therapies using CAP in combination with other existing approaches.

## Materials and Methods

### Biosafety

All experiments with live HIV-1 were performed under the BSL-2 conditions.

### Cell lines, primary cells, HIV constructs, and infection

Plasma from uninfected donors was obtained from a commercial source (New York Blood Center, www.nybloodcenter.org), and donor identity and identifiable characteristics were unknown to investigators. Therefore, per NIH definition, this research does not qualify as research on human subjects. Primary monocytes were isolated from plasma as described [6] and plated in the 24-wells primary culture plates (1 x 10^6^ cells per well). Cells were cultured for ~7 days in RPMI media with 10% of human serum supplemented with M-CSF (2 ng/ml). Cell confluence was ~ 40–60%.

Macrophage-tropic HIV-1 ADA [7] was obtained through the NIH AIDS Reagent Program (Cat. # 416), propagated on primary MDM and kept frozen at -70°C. To prepare VSV-G-pseudotyped HIV-1 virions, human embryonic kidney (HEK) 293T/17 cell line [8, 9] was used. The cell line was purchased from ATCC (Cat. # CRL-11268^™^) in 2005, aliquoted and stored frozen. Cells were co-transfected with pNL4-3 [10] and pHEF-VSVG [11]. Culture supernatants collected 96 h after transfection were filtered through a 0.22 μm filter. CAP-treated macrophages were infected with HIV-1 ADA (R5 strain) or VSV-G-pseudotyped HIV-1 LAI (X4 strain) at 5 x 10^5^ cpm of RT activity/1x10^6^ cells for 3 hours, followed by 3 washes with 1 x PBS. Infected cells were cultivated in fresh complete medium; every 3–4 days, half of the medium was changed and checked for RT activity.

### CAP treatment

**Fig 1:**
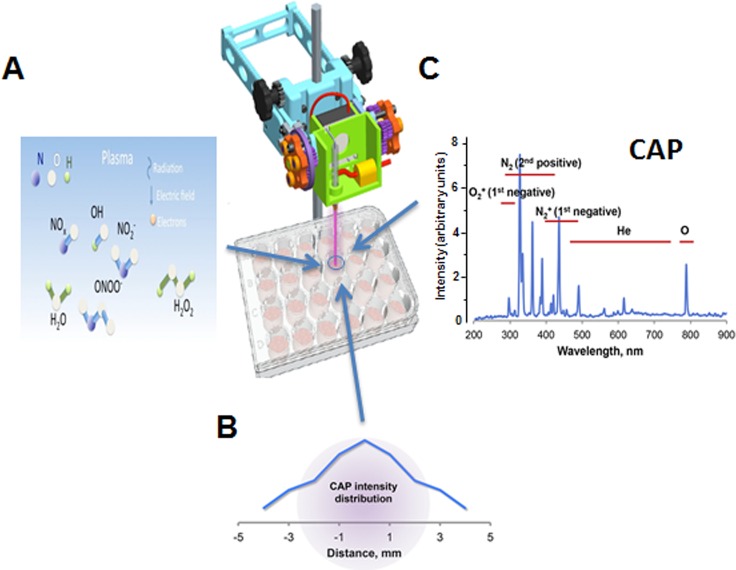
Characteristics of the CAP device. A. Experimental setup of CAP treatment. Reactive oxygen and nitrogen species in the plasma stream are shown. B. CAP intensity distribution in the jet beam at the well surface. C. Optical emission spectrum at the jet’s end characterizes irradiation from the entire length of the plasma column (CAP).

The CAP jet is the dielectric barrier discharge device shown in Fig 1A. The working parameters were: the output voltage was 4.5 kV in helium, the frequency ~13 kHz, the gas flow rate ~10 L/min. The distance between the jet outlet and culture plate was kept about 20 mm. The average jet dose at this distance was ~ 0.8 J/sec x cm^2^. The CAP beam diameter was about 8 mm, and intensity distribution within the beam at the well surface is shown in Fig 1B. The helium beam was used as a control for all experiments. We checked the surface temperature during irradiation using non-coupled infrared thermocouple, as described in our previous reports [12, 13]. The temperature was maintained at 37°C, and no temperature increases were observed during or after CAP treatment. Cell culture medium was changed right after CAP treatment to avoid medium effects on cells.

### MTT Assay

Cells were incubated with 7 mg/ml of Thiazolyl Blue Tetrazolium Bromide for 4 hours at 37°C. After removal of the supernatant and washing with PBS, acidic isopropanol solution (absolute isopropanol with 0.1 N HCl) was added. The results were read at 570 nm on an ELISA reader.

### Reverse Transcriptase Assay

10 μL of the medium from infected cultures was mixed with 10 μL of 5 x RT buffer (250 mM Tris-HCl, pH 8.0, 5 mM DTT, 25 mM MgCl_2_, 100 mM KCl), 10 μL of poly-A (1 U/mL), 10 μL of poly-dT (1 U/mL), 2 μL of [^3^H]TTP (40 μCi/mL), 3 μl H_2_O and 5 μL Triton 1%. Following incubation for 2 h at 37°C, 5 μL of the mixture was spotted on DEAE Filtermat paper, dried, washed in 5% Na_2_HPO_4_, and rinsed 3 times in H_2_O and 1 time in 70% EtOH. Samples were dried again, sealed in the sample bags, and counted on a Betaplate counter.

### Live Cell Staining

Macrophage cultures were washed on ice with 1 x PBS followed by addition of 10 mM EDTA for 5–10 min at room temperature. Cells were aspirated into the microfuge tubes, washed with 1 x PBS twice, and resuspended in blocking buffer for Human Fc receptor (Bioscience) for 30–60 min on ice. After incubation with Fc block, without washing, antibodies were added for 30 min on ice. Cells were then washed, resuspended in 2% FBS and stained. Fixable Viability Dye eFluor®450 (Bioscience), a viability dye, was used at a 1:1,000 dilution to irreversibly label dead cells. CCR5 was stained using anti-human CD195 (BD Pharmigen), CD4 –using anti-human Leu-3 T4 (Bioscience), with isotype controls mouse IgG2a K (BD Pharmigen) and mouse IgG2b K (Bioscience), respectively.

### Flow cytometry

Flow cytometry analyses were performed on a FACSCalibur DxP8 at the GWU Flow Cytometry Core Facility. The analyzer is equipped with three lasers (providing excitation wavelengths of 488, 637 and 407 nm) and eight detectors for fluorescence.

### Data Analysis

Statistical analysis of data was performed by means of InStat 3 software (Graphpad Software). The Kolmogorov-Smirnov test was applied to test for Gaussian distribution. Depending on the sample size, parametric or non-parametric analysis was used to determine the means and standard deviation values. The differences between groups were determined by two tail unpaired t-tests. Data were considered significant for *p*-values *<* 0.05.

## Results and Discussion

**Fig 2:**
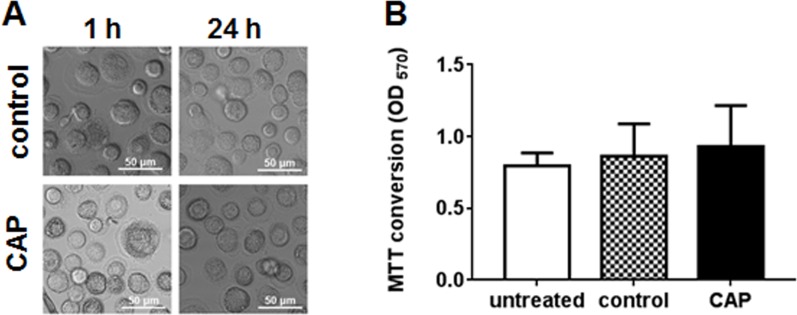
Analysis of CAP cytotoxicity. A. Morphology of MDM after CAP treatment. Macrophages in the CAP-treated area were imaged 1 h and 24 h after treatment (45 sec at 4.5 kV). B. Cell viability was assessed by MTT assay. MTT conversion to formazan was measured at OD_570_.

CAP treatment was applied to cells using 3 shots (45 sec at 4.5 kV) per well in a 24-well plate. To determine the percentage of cells affected by CAP treatment we measured the spectrum across the plasma jet flow. The special plastic holder for spectrometer probe was built and used for 1 mm step measurements. The O_2_ (First Negative) peak at 327 nm (the highest intensity in the spectrum, see Fig 1C) was chosen to evaluate the jet performance. The full width of the distribution (the jet beam diameter) was found to be ~8 mm. Thus, for the well diameter of 15.6 mm, 3 shots cover 80% of the well area, indicating that 80% of cells are treated. Given that over 80% of intensity is localized to the area with 4 mm diameter (new Fig 1B), about 25% of affected cells received maximal treatment. In total, 20% of cells on the plate received maximal treatment, 60% got intermediate dose, and 20% were not treated at all. The cold plasma jet system operated at atmospheric pressure can produce chemically active species, particularly oxygen and nitrogen atoms [14, 15]. To characterize the emission spectrum of our jet, we employed optical emission spectroscopy (OES) over a wide range of wavelengths from 200 nm to 900 nm with an optical emission spectroscope (SV 2100, K-MAC). The dominant emission lines illustrate the presence of excited oxygen ions (O_2_^+^) at 300–350 nm and atomic oxygen (O) at 800–844 nm (Fig 1C). We also detected NO species in the 200–300 nm range, and reactive nitrogen species N_2_ (N_2_ second positive system) and ionized nitrogen molecules N_2_^+^ (N_2_^+^ first negative system) in the ranges of 300–420 nm and 400–500 nm, respectively (Fig 1C). In addition, many other reactive species, such as OH, H_2_O_2_, ONOO^-^ and others (shown schematically in Fig 1A), are formed in the liquid phase in the cell culture medium [15]. However, despite abundance of these bioactive molecules, CAP treatment was not cytotoxic to uninfected macrophages, as evidenced by unchanged cell morphology (Fig 2A) and metabolic activity measured by MTT assay (Fig 2B). Previously, CAP treatment using the same conditions was shown to be non-toxic to murine primary keratinocytes and epithelial cell lines [13].

**Fig 3:**
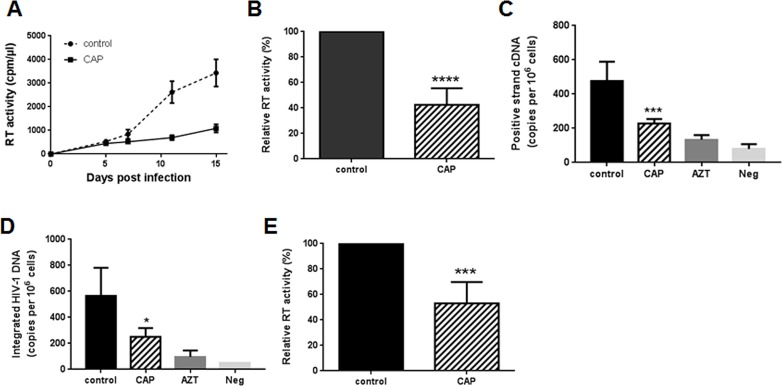
CAP effects on HIV-1 replication. A. Monocyte-derived macrophages treated with CAP or helium (control) were infected with HIV-1 ADA and viral replication was monitored for 15 days by RT activity in the culture supernatant. B. Results (mean±SD) are presented for RT analysis on day 15 after infection (performed as in panel A) for 7 different donors. ****p<0.0001 by Student’s unpaired two-tail *t*-test. C. MDM infected with HIV-1 ADA as in A were analyzed 4 h post infection by qPCR for positive-strand cDNA. Cells treated with AZT (3 μM) and uninfected cells are shown as negative controls. Results are presented as mean±SD for four independent infections with cells from one representative donor. ***p = 0.0004 by Student’s unpaired two-tail *t*-test. D. HIV-infected MDM were analyzed 48 h post-infection by Alu-GAG qPCR for integrated proviral DNA. Results are presented as mean±SD for four independent infections with cells from one representative donor. *p = 0.0494 by Student’s unpaired two-tail *t*-test. E. HIV-1 ADA was treated with CAP or helium (control) and used to infect MDM. Virus infection was assessed by measuring reverse transcriptase activity in culture supernatant on day 10–15 post-infection. Results (mean±SD) are presented for 6 experiments with MDM from independent donors. ***p<0.001 by Student’s unpaired two-tail *t*-test.

Analysis of HIV-1 replication in CAP-treated cells demonstrated a 3–4 fold reduction in RT activity measured in culture supernatants over a period of 14 days (Fig 3A). This effect was reproduced with MDM from 7 donors (Fig 3B). Such inhibitory effect could be due either to the CAP effect on cells, which made them resistant to infection, or to the effect on the virus produced in treated cells, which reduced its infectivity for the next round of infection. Of course, it was possible that both these scenarios were at work.

To address the mechanism of CAP activity, we first analyzed HIV reverse transcription products in cells treated with CAP prior to infection. This analysis demonstrated that both positive strand cDNA products of reverse transcription (Fig 3C) and integrated proviral DNA (Fig 3D) were reduced in CAP-treated cells by 2.5-fold and 3-fold, respectively. This result suggested that viral entry or reverse transcription was defective in cells pre-treated with CAP.

We next tested whether treatment of the virus, rather than of the cells, with CAP reduced infection. Results presented in Fig 3E demonstrate that treatment of the virus suspension with CAP significantly reduced the ability of this virus to establish infection in MDM. These results have been reproduced with MDM from 6 different donors. This effect of CAP could be due to damage to the viral envelope, which prevented virus-cell fusion, or destruction of the viral capsid leading to impairment of reverse transcription. Future studies would be needed to define the exact mechanism involved in this activity of CAP.

**Fig 4:**
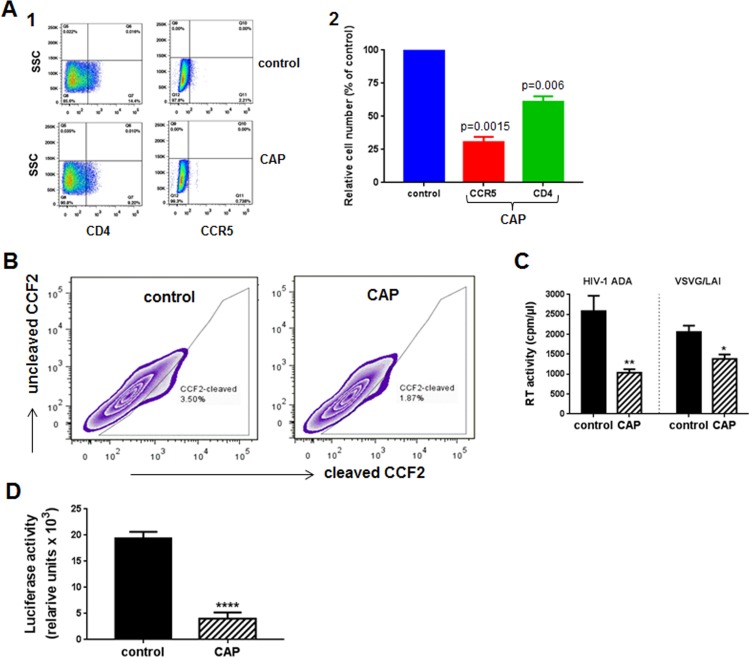
Mechanisms of anti-HIV activity of CAP treatment. A1. MDM treated with CAP or helium (control) were analyzed by flow cytometry for expression of CCR5 and CD4. A2. Results are presented as mean ± SEM for analysis performed for MDM from two donors. P values (relative to control) were calculated using Student’s unpaired two-tail *t*-test. B. Fusion between HIV-1 and MDM was analyzed by fluorescence resonance energy transfer-based fusion assay. Cleavage of CCF2 represents virus-cell fusion. C. MDM treated with CAP or helium (control) were infected in triplicate wells with HIV-1 ADA (R5 virus) or VSV-G-psudotyped HIV-1 NL4-3 (X4 virus), and viral production was measured on day 7 by RT activity. Results are presented as mean ± SD. *p = 0.0037, **p = 0.027 relative to control, calculated using Student’s unpaired two-tail *t*-test. D. MDM treated with CAP or helium (control) were infected with HIV-1 ADA and incubated for 21 days. Virus was collected, adjusted to the same RT activity by dilution, and used to infect indicator TZM-bl cells. Results are presented as mean ± SD for 5 independent replicates. ****p<0.0001 by Student’s unpaired two-tail *t*-test.

Reduced efficiency of reverse transcription and integration in cells treated with CAP could be due to inhibited entry of the virus, impaired reverse transcription and integration, or both. To investigate CAP effects on viral entry, we analyzed expression of HIV receptors on CAP-treated macrophages. This analysis revealed that expression of both CD4 and CCR5 receptors was reduced (Fig 4A1). Comparison of two experiments with MDM from different donors demonstrated that this effect was reproducible and significant: CCR5 expression was reduced by 70% and CD4 –by 30% (Fig 4A2). Reduced expression of HIV receptors was expected to translate to reduced fusion between HIV-1 and target cell. We analyzed HIV-1-cell fusion using the fluorescence resonance energy transfer-based fusion assay [16]. HEK 293T cells were co-transfected with HIV-1 NL4-3 molecular clone and BlaM-Vpr, virions were collected by centrifugation and used to infect CAP-treated MDM loaded with CCF2-AM. Cells were analyzed by flow cytometry, using excitation at 409 nm and measuring emission at 520 nm (uncleaved CCF2) and 450 nm (CCF2 cleaved by BlaM). Percentage of cells with cleaved CCF2 reflects the efficiency of fusion. We found fusion to be reduced by about 40% (Fig 4B). This result was reproduced with cells from a different donor. These results indicate that CAP treatment modifies the cell membrane making them less susceptible to HIV infection. It should be noted that the effect measured in the fusion assay (Fig 4B) represents the whole population of treated cells, where 20% of cells remained untreated and 60% received sub-optimal dose (Fig 1B). Therefore, the actual inhibitory effect of CAP is likely more potent.

To determine whether changes in HIV receptors expression are the only reason for reduced susceptibility of CAP-treated macrophages to HIV infection, we analyzed infection by VSV-G-pseudotyped NL4-3 virus, which enters target cells via endocytosis thus bypassing the HIV receptor-mediated fusion with the target cell plasma membrane during entry. This virus goes through only one cycle of replication in macrophages. Surprisingly, replication of VSV-G-pseudotyped HIV-1 was also significantly reduced in CAP-treated cells (Fig 4C). This result suggests that, in addition to the effect on HIV receptors, CAP treatment affected cellular factors involved in post-entry steps of HIV replication, e.g. nucleotides required for reverse transcription. Given that CAP treatment was not associated with cytotoxicity, it appears that affected cellular factors are not essential for macrophage survival, at least during the period needed for HIV infection to proceed through one cycle of replication in macrophages.

Next, using indicator TZM-bl cells, we analyzed infectivity of HIV virions collected from CAP-treated macrophages. This analysis revealed a 4-fold reduction in infectivity of such virus relative to virus collected from control (helium-treated) cells (Fig 4D). This result suggests that virus produced in CAP-treated cells is impaired during the next cycle of infection. Given that cells were pre-treated with CAP, so CAP did not contact nascent virions, the observed impairment is consistent with the observed inhibition of infection by VSV-G-pseudotyped virus and was likely due to the effect on nascent virions of active radicals, reactive oxygen species, and other bioactive molecules produced in CAP-treated cells (Fig 1B).

Taken together, results of this study demonstrate that CAP-induced suppression of HIV replication involves at least three major mechanisms: reduction of expression of HIV receptors on target cells, which leads to reduced virus-cell fusion and virus entry, damage of cellular factors required for post-entry events of HIV replication and reduction of infectivity of the produced virions, most likely via an indirect mechanism mediated by active radicals formed as a result of CAP treatment, and direct damage of the virions by CAP. The contribution of each of these mechanisms may vary during the course of HIV spreading infection, e.g. initially the fusion inhibition may dominate, whereas later the effect on virions may be the major inhibitory mechanism. This interpretation is supported by a similarity between the inhibitory effects of CAP on HIV replication at day 12 post-infection (about 4-fold, Fig 3A) and infectivity of virions collected after 21 days of infection (4-fold, Fig 4D). Molecular details of the mechanisms involved in these interesting activities of cold plasma await to be determined.
